# Systematic survey of the function of ROP regulators and effectors during tip growth in the moss *Physcomitrella patens*

**DOI:** 10.1093/jxb/ery376

**Published:** 2018-10-31

**Authors:** Carlisle Bascom, Graham M Burkart, Darren R Mallett, Jacquelyn E O’Sullivan, Alexis J Tomaszewski, Katherine Walsh, Magdalena Bezanilla

**Affiliations:** 1 Plant Biology Graduate Program, University of Massachusetts, Amherst, USA; 2 Department of Biological Sciences, Dartmouth College, Hanover NH, USA; 3 Biology Department, University of Massachusetts, Amherst, USA

**Keywords:** ROP, ROPGAP, ROPGEF, ROPGDI, REN, Spike, RNAi, *Physcomitrella patens*

## Abstract

Rho/Rac of plants (ROP) GTPases are plant-specific small GTPases that regulate cell morphology. ROP activity is controlled by several families of regulatory proteins. However, how these diverse regulators contribute to polarized growth remains understudied. In a system-wide approach, we used RNAi to silence each gene family of known ROP regulators in the juvenile tissues of the moss *Physcomitrella patens*. We found that the GTPase activating proteins, but not the ROP enhancers, are essential for tip growth. The guanine exchange factors (GEFs), which are comprised of ROPGEFs and Spikes, both contribute to growth. However, silencing Spikes results in less-polarized plants as compared to silencing ROPGEFs, suggesting that Spikes contribute more to establishing cell polarity. Silencing the single-gene family of guanine dissociation inhibitors also inhibits growth, resulting in small, unpolarized plants. In contrast, silencing the ROP effector ROP-interactive CRIB-containing (RIC) protein, which is encoded by a single gene, results in plants larger than the controls, suggesting that RIC functions to inhibit tip growth in moss. Taken together, this systematic loss-of-function survey provides insights into the function of ROP regulators during polarized growth.

## Teaser

Systematic loss of function studies reveal the influence that each family of Rho of plants (ROP) regulators has on tip growth in the moss, *Physcomitrella patens*.

## Introduction

Rho/Rac of plants (ROPs) are a plant-specific family of small GTPases. ROPs act as molecular switches: they associate with the plasma membrane, are active in a GTP-bound state, and after hydrolysis of GTP to GDP they are inactive. In their active state, ROPs have been shown to modulate many cellular processes (Craddock *et al.*, 2012; Feiguelman *et al.*, 2018). In seed plants, the ROP gene family is comprised of multiple genes. For example, in *Arabidopsis thaliana*, there are 11 ROP genes that can be further divided into four groups (Zheng and Yang, 2000; Christensen *et al.*, 2003; Eklund *et al.*, 2010). Within each group, the genes have been implicated in similar biological processes. For example, the five Arabidopsis ROP genes in group IV play important roles in tip growth (Eklund *et al.*, 2010).

In the moss *Physcomitrella patens*, there are only four ROP genes, encoding for nearly identical proteins (Eklund *et al.*, 2010). In fact, there is more sequence diversity among the Arabidopsis ROPs than among the four in *P. patens*. In *P. patens*, ROPs are essential for polarized growth, and they affect cell wall deposition, cell adhesion, and actin dynamics (Burkart *et al.*, 2015). A single ROP gene is sufficient for polarized growth (Burkart *et al.*, 2015). Overall, the levels of ROP mRNA expression are tightly regulated, and modest reductions result in reduced growth (Burkart *et al.*, 2015).

As GTPases, ROPs function to activate or inactivate downstream effectors. In seed plants, a number of proteins have been identified as ROP effectors (Wu *et al.*, 2001). Using sequence similarity, it has only been possible to identify one ROP effector in *P. patens*, namely ROP-interactive CRIB-containing (RIC) proteins. In Arabidopsis, there are 11 RIC genes (Eklund *et al.*, 2010), some of which have been shown to be specific effectors for a subset of ROP proteins (Wu *et al.*, 2001). RICs mediate different cellular processes, and a single RIC protein can have differing functions depending on the cell type. For instance, RIC1 is thought to manipulate F-actin in pollen tubes to control polarity and growth but to promote severing of microtubules in leaf pavement cells (Fu *et al.*, 2005; Zhou *et al.*, 2015).

ROP GTPase activity is directly regulated by proteins that remove ROP from the plasma membrane as well as by proteins that alter the GTP-bound state. Guanine dissociation inhibitors (GDIs) remove GDP-bound GTPases from the plasma membrane by sequestering the prenyl modification and forming cytosolic heterodimers (Koch *et al.*, 1997; Dovas and Couchman, 2005). Plants have a single family of ROPGDIs with a well-conserved immunoglobulin-like (IG) domain that is responsible for sequestering the prenyl moiety. GTPase-activating proteins (GAPs) activate the intrinsic GTP hydrolysis, resulting in GDP-bound protein, which is the inactive form. In plants, there are two families of GAPs that contain the conserved RhoGAP domain: ROPGAPs and ROP enhancers (RENs). ROPGAPs are relatively small, ~430 amino acids, with a CRIB domain near their N-termini. In contrast, REN proteins are large, ~830 amino acids, and have a number of other domains including PH domains, and two putative coiled-coil interaction domains (Hwang *et al.*, 2008; Eklund *et al.*, 2010). To return ROP to the active confirmation, the GDP must be removed and replaced by GTP. This activity is mediated by guanine nucleotide exchange factors (GEFs). Plants also have two families of GEFs: ROPGEFs and Spikes. Spikes are distantly related to the CZH RhoGEFS found in animals and fungi (Basu *et al.*, 2008).

In this study, we used gene silencing to test whether *P. patens* RIC is involved in tip growth during the establishment of the juvenile moss plant, which is comprised of filamentous tissue that expands exclusively by tip growth. Furthermore, to investigate the role each family of ROP regulators has in tip growth in moss protonemata, we conducted a systematic loss-of-function of analysis of each of the ROP regulator gene families.

## Materials and methods

### Plant material

*Physcomitrella patens* tissue was kept in the juvenile growth state with weekly propagation. Briefly, 1-week-old tissue was lightly homogenized in water and pipetted onto a 10-cm Petri dish containing 25 ml of standard solid growth media (described by Wu and Bezanilla, 2014) covered by permeable cellophane. Plants were grown under 85 μmol photons m^–2^ s^–1^ provided by fluorescent bulbs in long-day conditions (16/8 h light/dark). RNAi assays were performed in a line stably expressing NLS-GFP-GUS (NLS4) (Bezanilla, 2003; Bezanilla *et al.*, 2005). To measure actin dynamics in GDI-silenced plants, we used a line stably expressing NLS-GFP-GUS as well as Lifeact-mEGFP (NLS4/Lifeact-mEGFP) (Vidali *et al.*, 2010).

### Plasmid construction

RNAi constructs were generated by amplifying regions of the coding or untranslated sequences of target genes from genomic DNA or cDNA isolated from 7-d-old protonemal RNA. An entry clone of the amplicon was generated using the pENTR/D-Topo vector (Invitrogen). The final RNAi construct was constructed by performing an LR clonase (Invitrogen) reaction of the entry clone with the RNAi destination vector pUGGi (Bezanilla *et al.*, 2005; Vidali *et al.*, 2007). When multiple genes were targeted with the same construct, a combination of two approaches was used to generate the entry clones: amplicons were assembled with a technique described previously (Vidali *et al.*, 2007) that uses a combination of ligation and PCR amplification; and/or were ligated together using conventional molecular genetic techniques. Each entry clone construction is detailed below and the primers used to generate all amplicons are listed in Supplementary Table S1 at *JXB* online. All gene identifications listed are from version 3.3 of the *P. patens* genome (Lang *et al.*, 2018).

#### RIC

An amplicon containing 128 bp of the 5′-untranslated region (UTR) and the first 466 bp of the coding sequence of RIC (Pp3c12_19130V3.1) was amplified by PCR and transferred to the pENTR/D-Topo vector.

#### ROPGAP

Regions of the coding sequences for *ROPGAP3* (Pp3c13_4010V3.1) and *ROPGAP1* (Pp3c4_16800V3.1) were amplified, stitched together with a BamHI site, and subsequently cloned into the pENTR/D-Topo vector. Regions of the coding sequences for *ROPGAP4* (505–726 of coding sequence of Pp3c4_24980V3.1) and *ROPGAP5* (511–739 of coding sequence of Pp3c26_4490V3.1) were amplified and stitched together with a Bsu36I site. The ROPGAP4/ROPGAP5 amplicon was engineered to contain BamHI sites on the 5′ and 3′ ends and these sites were used to clone this amplicon in between *ROPGAP3* (772–987 of coding sequence) and *ROPGAP1* (823–1040 of coding sequence). Regions of the coding sequences for *ROPGAP2* (623–894 of coding sequence of Pp3c3_5940V3.1) and *ROPGAP6* (463–882 of coding sequence of Pp3c26_5960V3.1) were amplified and stitched together with an EcoRI site. The *ROPGAP2*/*ROPGAP6* amplicon was cloned into the pGEM T-Easy vector (Promega). NotI was used to release the *ROPGAP2*/*ROPGAP6* fragment from pGEM T-Easy and subsequently ligated into a NotI site upstream of *ROPGAP3*. The final entry clone in the pENTR/D-Topo vector contained the following regions of sequence and restriction enzyme sites: NotI-*ROPGAP2*-EcoRI-*ROPGAP6*-NotI-*ROPGAP3*-BamHI-*ROPGAP4*-Bsu36I-*ROPGAP5*-BamHI-*ROPGAP1*.

#### ROPGEF

Regions of the coding sequences for *ROPGEF2* (985–1211 of coding sequence of Pp3c10_9910V3.1) and *ROPGEF4* (691–898 of coding sequence of Pp3c2_28420V3.1) were amplified, stitched together with a BamHI site, and subsequently cloned into the pENTR/D-Topo vector. Regions of the coding sequences for *ROPGEF6* (963–1192 of coding sequence of Pp3c1_36410V3.1) and *ROPGEF5* (716–940 of coding sequence of Pp3c14_22480V3.1) were amplified and stitched together with a Bsu36I site. The *ROPGEF6*/*ROPGEF5* amplicon was engineered to contain BamHI sites on the 5′ and 3′ ends and these sites were used to clone this amplicon in between *ROPGEF2* and *ROPGEF4*. Regions of the coding sequences for *ROPGEF1* (749–1028 of coding sequence of Pp3c2_4460V3.1) and *ROPGEF3* (493–747 of coding sequence of Pp3c1_20V3.1) were amplified and stitched together with BamHI site. The *ROPGEF1*/*ROPGEF3* amplicon was engineered to contain NotI sites on the 5′ and 3′ ends and these sites were used to clone this amplicon into a NotI site in the pENTR/D-Topo vector. The final entry clone in pENTR/D-Topo contained the following regions of sequence and restriction enzyme sites: *ROPGEF1*-BamHI-*ROPGEF3*-NotI-*ROPGEF2*-BamHI-*ROPGEF6*-Bsu36I-*ROPGEF5*-BamHI-*ROPGEF4*.

#### *Spike (SPK*)

Regions of the coding sequences for *SPK2* (1–380 of coding sequence of Pp3c9_15160V3.1) and *SPK5* (4458–4706 of coding sequence of Pp3c3_15370V3.1) were amplified, stitched together with a BamHI site, and subsequently cloned into the pENTR/D-Topo vector. A region of the coding sequence for *SPK4* (5295–5545 of coding sequence of Pp3c1_25190V3.1) was amplified and engineered to contain BamHI sites on the 5′ and 3′ ends. These sites were used to clone this amplicon in between *SPK2* and *SPK5*. Regions of the coding sequences for *SPK1* (5333–5566 of coding sequence of Pp3c15_8680V3.1) and *SPK6* (1–380 of coding sequence of Pp3c10_16640V3.1) were amplified, stitched together with a BamHI site, and subsequently cloned into the pENTR/D-Topo vector. A region of the coding sequence for *SPK3* (4386–4831 of coding sequence of Pp3c4_25310V3.1) was amplified and engineered to contain BamHI sites on the 5′ and 3′ ends. These sites were used to clone this amplicon in between *SPK1* and *SPK6*. The *SPK1*/*SPK3*/*SPK6* amplicon was engineered to contain NotI sites on the 5′ and 3′ ends and these sites were used to clone this amplicon into a NotI site in the pENTR/D-Topo vector. The final entry clone in pENTR/D-Topo contained the following regions of sequence and restriction enzyme sites: *SPK1*-BamHI-*SPK3*-BamHI-*SPK6*-NotI-*SPK2*-BamHI-*SPK4*-BamHI-*SPK5*.

#### REN

The first 423 bp of the coding sequence of *REN* (Pp3c9_17460V3.1) was amplified by PCR and transferred to the pENTR/D-Topo vector.

#### ROPGDI

The ROPGDI coding sequence RNAi construct used a 408-bp fragment of *ROPGDI2* (Pp3c10_19740V3.2) to target all four ROPGDIs (352–759 of coding sequence). This fragment was amplified and transferred into the pENTR/D-Topo vector. To generate the 5′-UTR construct, fragments from *ROPGDI1* (Pp3c3_32980V3.2), *ROPGDI2*, and *ROPGDI3* (Pp3c10_19650V3.1) 5′-UTRs were amplified from cDNA isolated from 7-d-old protonemata, introducing restriction enzyme sites via the primers. Fragments of the *ROPGDI1* 5′-UTR (–306 to 0 upstream of the start codon) were ligated to a fragment of the *ROPGDI2* 5′-UTR (–300 to 0 upstream of the start codon) via BamHI. An amplicon from this ligation was cloned into the pENTR/D-Topo vector. A fragment of the *ROPGDI3* 5′-UTR (–300 to 0 upstream of the start codon) was amplified separately and transferred into the pENTR/D-Topo vector. The 3′ end of the *ROPGDI2* UTR and the 5′ end of *ROPGDI3* UTR had EcoRI sites introduced via PCR. To create a *ROPGDI1*,2,3 5′-UTR entry clone, both *ROPGDI1*,*2* pENTR and *ROPGDI3* pENTR were digested with EcoRI and AscI. The *ROPGDI3* 5′-UTR drop-out was then ligated into the *ROPGDI1*,*2* linearized plasmid.

#### ROPGDI expression plasmids

*ROPGDI1* and *ROPGDI4* (Pp3c3_33010V3.1) encode for the same protein, referred to hereafter as ROPGDI1/4, and *ROPGDI2* and *ROPGDI3* also encode for the same protein, referred to hereafter as ROPGDI2/3. To express ROPGDI1/4 we amplified *ROPGDI4*, and to express ROPGDI2/3 we amplified *ROPGDI2*. Full-length coding sequences were amplified from cDNA isolated from 7-d-old protonemal tissue using the primers listed in Supplementary Table S1. Amplicons were transferred into the pENRT/D-Topo vector. Due to significant sequence identity, the same reverse primer was used to amplify both *ROPGDI2* and *ROPGDI4*. The binding region of the reverse primer differs by a single nucleotide between *ROPGDI2* and *ROPGDI4*, and therefore this primer changed the 756th nucleotide of GDI4 from a T to a C, introducing a silent mutation. The coding sequences were transferred to the pTH Ubi-Gate vector, using an LR reaction. pTH Ubi-Gate (Vidali *et al.*, 2007) drives expression of the coding sequence via a maize ubiquitin promoter, and confers hygromycin resistance to the plants.

To generate *ROPGDI2*-mEGFP, Gateway attachment sites were designed into primers (Supplementary Table S1) used to amplify *ROPGDI2* coding sequence without the stop codon. The *ROPGDI2*_L1R5_pENT plasmid was produced via a Gateway BP Clonase reaction. A multi-site LR reaction was used to introduce mEGFP downstream of *ROPGDI2* into pTK Ubi-Gate (Wu and Bezanilla, 2014). pTK UBi-Gate has the same backbone as pTH Ubi-Gate, but contains a neomycin-resistance cassette.

#### GFP tagging at the ROPGDI3 locus

Generating a *ROPGDI3*-mEGFP knock-in construct required cloning two regions of homology, namely the 5′ region of sequence upstream of the stop codon, and the 3′ region of sequence downstream of the stop codon. The 5′ (1003 bp upstream of the stop codon) and 3′ (a 999 bp region 1119 bp downstream of the start codon) homology regions were amplified from genomic DNA using the primers listed in Supplementary Table S1. BP Clonase reactions transferred these amplicons into their respective entry clones, which were sequenced. The final construct was generated with a multi-site LR clonase reaction using the pGEM-Gate (Vidali *et al.*, 2009*b*) destination vector, the entry clones described above, and two additional entry clones, L5L4_mEGFP and R4R3_ZeocinR.

#### Yeast two-hybrid

*Rop3* (Pp3c1_21550V3.1), *Rop4* (Pp3c10_4950V3.1), *ROPGAP1*, *ROPGAP2*, *ROPGAP3*, *ROPGAP5*, *ROPGAP6*, and *REN* were amplified with the primers indicated in Supplementary Table S1 from cDNA isolated from 7-d-old protonemal tissue. Except where noted below, full-length coding sequences were amplified. The *ROPGAP2* amplicon encoded amino acids 131–542; the *ROPGAP3* amplicon encoded amino acids 129–541; the *ROPGAP5* amplicon encoded amino acids 49–455. All ROPGAP amplicons spanned the conserved CRIB and RhoGAP domains. To generate the constitutively active (G15V) and dominant negative (T20N) forms of *Rop3* and *Rop4*, we performed site-directed mutagenesis (Weiner *et al.*, 1994) using the primers listed in Supplementary Table S1. All amplicons were transferred to the pENTR/D-Topo vector following the manufacturer’s instructions.

The Matchmaker Gold Yeast Two-Hybrid system (Takara Bio) was used for these studies. To make the bait (pGBKT7) and prey (pGADT7) plasmids compatible with Gateway cloning, we transferred the Gateway cassette using conventional cloning into the bait and prey plasmids to generate pGBKT7-Gate and pGADT7-Gate, respectively. ROPs were cloned into the bait plasmid and the ROPGAPs and REN were cloned into the prey plasmid using an LR clonase reaction. Two hybrid interaction studies were performed following the manufacturer’s recommendations.

### Transformation and growth assays

Protoplasts were transformed using a PEG-mediated protocol described by Schaefer *et al.* (1991) and RNAi growth assays were performed as described previously (Vidali *et al.*, 2007; Augustine *et al.*, 2008). Protoplasts were generated from 7-d-old tissue homogenized using Driselase (Sigma). Transformations were performed with 30 μg of plasmid, unless otherwise indicated. After transformation, protoplasts were plated onto regeneration media (described in Wu and Bezanilla, 2014). The RNAi plasmids contained a hygromycin-resistance cassette, and therefore regenerating plants were transferred to standard growth media supplemented with 15 μg ml^–1^ hygromycin 4 d after transformation. Plants were imaged 7 d after transformation using a fluorescence stereomicroscope (Leica MZ16FA) via a color camera (Leica DF300X) with a GFP2 filter. Living plants were visually screened for active silencing of the NLS-GFP-GUS marker.

### Quantifying plant area and solidity

Image analysis was performed with ImageJ as described by Vidali *et al.* (2007). Briefly, the plant to be processed was selected with the Draw Polygon tool, and this region was copied to a new blank stack. Next, the threshold for the red channel (chlorophyll autofluorescence) was defined, and a mask was made of the threshold. Both the area and solidity were measured from this mask. Solidity is the ratio of plant area to the convex hull area of the plant. In all instances, the area was normalized to control conditions.

### Western blotting

To detect the GDI3-mEGFP fusion protein, 7-d-old tissue was ground to make protein extracts were. As controls, the wildtype and a transgenic line expressing cytosolic GFP were processed in parallel. After freezing in liquid nitrogen and grinding with a mortar and pestle, tissue powder was suspended in 300 μl extraction buffer with the following composition: 100 mM sodium phosphate, 10 mM DTT, 0.02 mg ml^–^ leupeptin, 1 mM PMSF, 20% glycerol, 5 mM EDTA, and a cOmplete Mini (Roche) protease inhibitor tablet. Samples of 5 μl from each extract were separated on a 10% polyacrylamide gel, and the separated proteins were transferred onto an Amersham Protran nitrocellulose membrane (GE Healthcare). The membrane was blocked for 1 h at room temperature and incubated overnight at 4 ºC with a primary antibody raised against GFP (Augustine *et al.*, 2008). The membrane was then washed and treated with a secondary antibody against rabbit for 1 h at 25 ºC. The membrane was again washed, developed with Clarity Western ECL Substrate (BioRad), and imaged with an ImageQuant LAS 500 (GE Healthcare).

### Variable angle epifluorescence microscopy and confocal imaging

Both variable angle epifluorescence microscopy (VAEM) and confocal imaging were carried out at an imaging facility at the University of Massachusetts, Amherst. A single inverted microscope body (Ti-E; Nikon Instruments) was equipped with a T-FL-TIRF arm and a CSU-X1 spinning disk confocal head (Yokogawa). The microscope was equipped with an iXON3 CCD camera (Andor Technology) for acquisition. Laser illumination at 488 nm with 525/50-nm emission filters was used to acquire mEGFP in both GDI3-mEGFP and Lifeact-mEGFP. The microscope was controlled by NIS Elements software (Nikon), and image processing was done in ImageJ.

### Measuring actin dynamics

The GDI UTR RNAi plasmid was transformed into the NLS4/Lifeact-mEGFP line as described previously (Vidali *et al.*, 2010). Both test and control transformants were selected for via hygromycin resistance. Seven-day-old plants were visually screened for active silencing (lack of nuclear GFP) using a fluorescent stereomicroscope (Leica DFC 300FX), and regions containing silenced plants were marked for VAEM. Silenced plants were transferred to an agar pad (Hoagland’s media with 1% agar) on a glass slide and then covered with a coverslip. ImageJ was used for post-acquisition processing. The correlation coefficient analysis was performed in MatLab as described by Vidali *et al.* (2010). Traces for individual cells were then averaged and plotted using KaleidaGraph.

## Results

### Silencing *RIC* has no effect on protonemal growth

For all silencing experiments, we used a transient RNAi assay that enabled rapid identification of silenced plants (Bezanilla *et al.*, 2005). In this assay, RNAi was performed in a moss line (NLS4) that stably expressed a nuclear-localized green fluorescent protein (GFP) fused to β-glucorinidase (GUS). The RNAi constructs contained inverted repeats of target gene sequences fused to GUS sequences, permitting simultaneous silencing of the target gene and the GFP-GUS fusion reporter. As a control, RNAi was performed with a construct that only contained GUS sequences. At 1 week after transformation, we acquired images of actively silenced plants identified by the lack of nuclear GFP fluorescence. Image acquisition was performed in a double-blind manner. We aimed to image 25 silenced plants per transformation, and control RNAi transformations generally yielded substantially more plants than this. However, sometimes it was not possible to image 25 plants for a particular RNAi construct, suggesting that the construct was incompatible with protoplast regeneration or plant viability. In these cases, all the silenced plants on the transformation plate were imaged. To quantify the effects of RNAi, we determined plant area as estimated from the chlorophyll autofluorescence, and plant solidity, which is the ratio of total plant area to convex hull area and reflects overall plant morphology. A solidity value of one represents an object without indentations, such as a solid circle, and values decrease as indentations in the object increase, which was more representative of control plants.

**Fig. 1.**
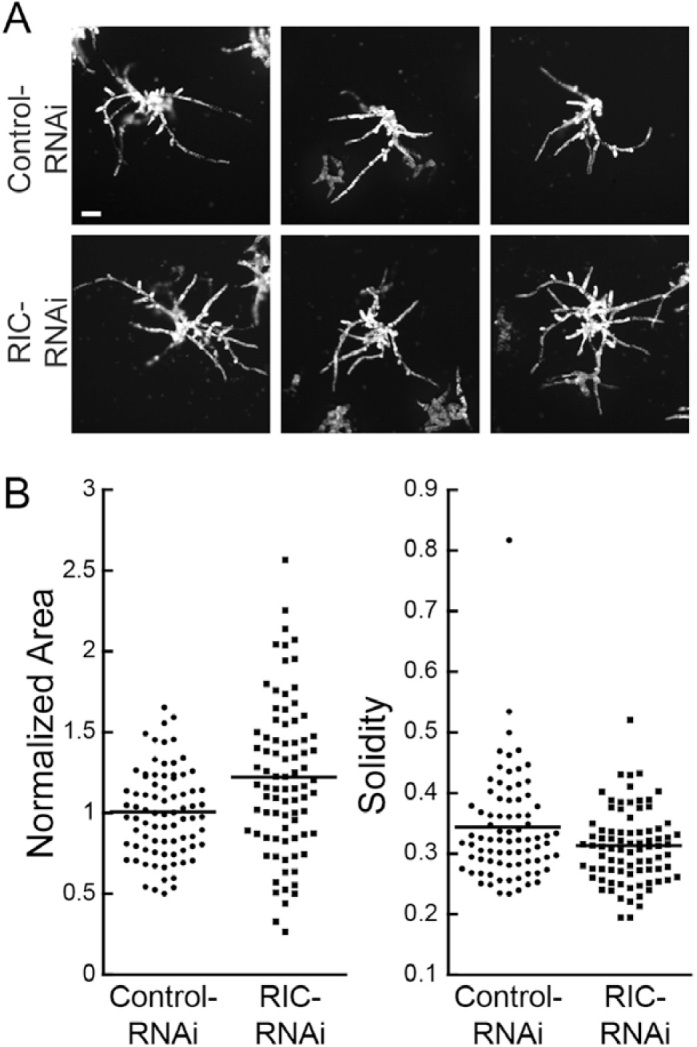
Transient RNAi of RIC does not impair growth. (A) Three representative images of the chlorophyll autofluorescence of 7-d-old plants transformed with either control- or *RIC*-RNAi constructs. The images are representative of average-sized plants taken from a pool of 80 control-RNAi and 83 *RIC*-RNAi plants across three transformations. The scale bar is 100 μm. (B) Quantification of plant area and solidity. Area is normalized to the average size of plants transformed with the control plasmid, and means are indicated by horizontal lines.

To silence RIC, which is encoded by a single gene in *P. patens*, we generated an RNAi construct that contained 594 bp of the *RIC* cDNA, namely 128 bp of the 5′-UTR followed by the first 466 bp of the coding sequence. We found that actively silenced plants transformed with the *RIC*-RNAi construct resembled control silenced plants (Fig. 1A). Interestingly, the *RIC*-RNAi plants were on average larger than control plants (Fig. 1B), suggesting that they may regenerate and/or grow faster than controls. Plant morphology as measured by solidity, on the other hand, was indistinguishable between RIC and control RNAi plants (Fig. 1B), suggesting that RIC does not play a significant role in regulating tip growth in protonemata.

### ROP regulators differentially impact polarized growth

Using sequence comparisons, only a single ROP effector, *RIC*, is easily identified in *P. patens*, whereas in contrast all plant families of ROP regulatory proteins can be readily identified (Eklund *et al.*, 2010). To determine which family contributes to regulating ROP function during polarized growth, we generated RNAi constructs that silenced each of the regulatory families and analysed the resulting phenotypes of 7-d-old plants.

**Fig. 2.**
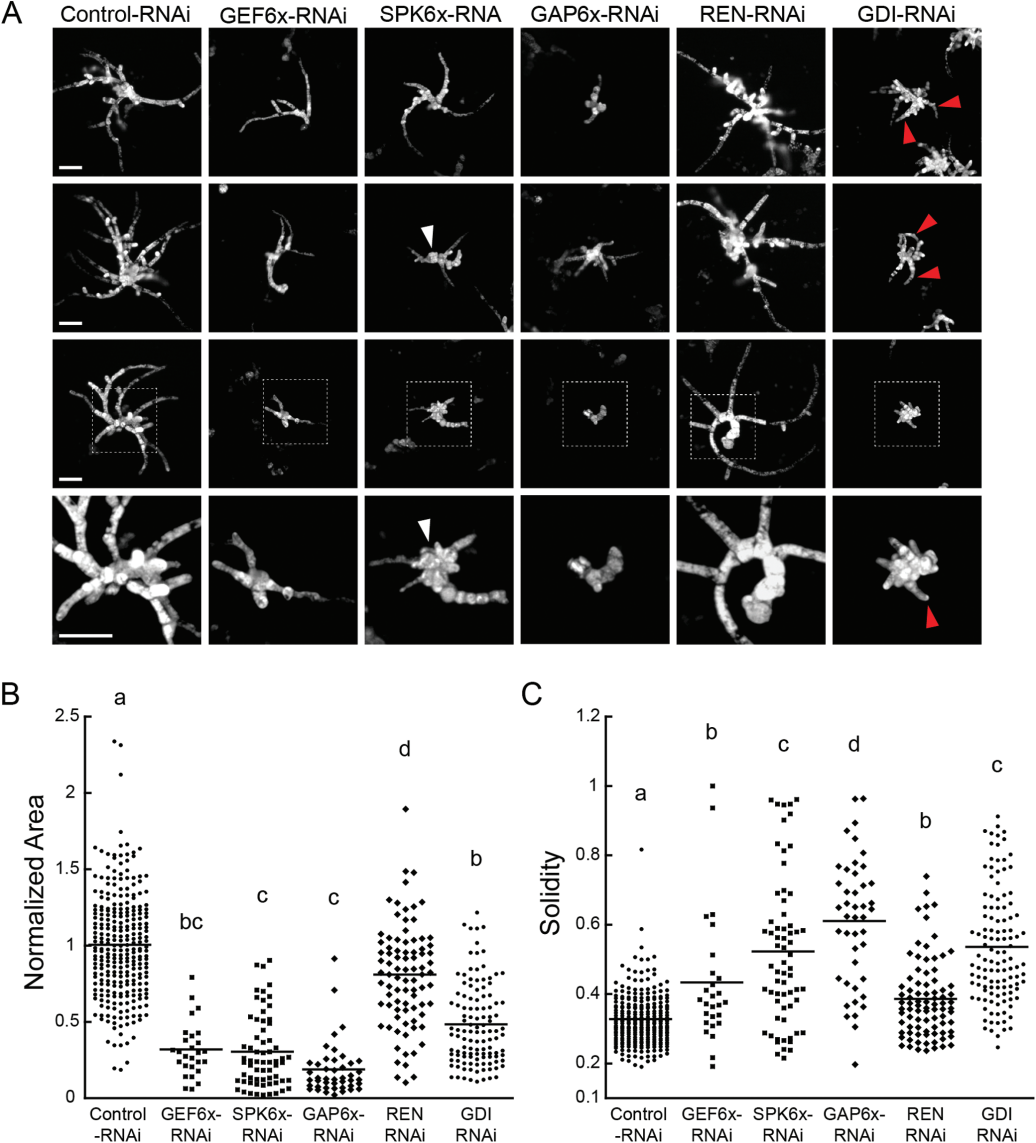
ROP regulators contribute differentially to polarized growth. (A) Representative images of chlorophyll autofluorescence from 7-d-old plants transformed with a construct silencing the indicated gene family. The images for each construct represent individual transformants and display areas similar to that of the average area for that construct (control, 275 plants from nine transformations; GEF6X-RNA, 27 plants from three transformations; *SPK6X*-RNAi, 65 plants from three transformations; *GAP6X*-RNAi, 44 plants from three transformations; *REN*-RNAi, 84 plants from three transformations; *GDI*-RNAi, 117 plants from three transformations). The regions indicated with boxes in the third row of images are shown in magnified view in the bottom row. Clusters of isotropic cells observed in *SPK6x*-RNAi plants and polarized extensions observed in *GDI*-RNAi plants are indicated with arrowheads. The scale bars for each row are 100 μm. (B) Quantification of plant area and solidity. Area is normalized to the average size of plants transformed with the control plasmid, and the means are indicated by horizontal lines. Different letters indicate groups with significantly different means as determined by ANOVA with a Tukey *post hoc* test (α=0.05).

GEFs exchange the GDP on ROP for GTP, thereby activating ROP. Therefore, reducing GEF activity should lead to less active ROP, and thus should result in a phenotype similar to loss of ROP function. There are 12 GEFs in *P. patens*: six ROPGEFs and six Spikes. Transformation with a construct that targeted the six ROPGEFs resulted in plants that were 69% smaller than controls (Fig. 2B). Transformation with a construct that targeted the six Spikes also resulted in plants of similar same size to those transformed with the GEF-silencing construct (Fig. 2B). However, there were significant differences with respect to the polarity and number of recovered *ROPGEF*-RNAi and *Spike*-RNAi plants.

*Spike*-RNAi, which yielded 22 plants per transformation, often had a cluster of globular cells near the initial protoplast (Fig. 2A) that closely resembled *ROP*-RNAi cells (Burkart *et al.*, 2015). In addition to the central globular cells, each plant often had a few polarized extensions. We found that *Spike*-RNAi plants had on average 3.25 ± 2.6 polarized extensions per plant (*n*=65 plants). This mixed morphology was also reflected in solidity values that were higher than the control (Fig. 2C), but substantially lower than the *ROP*-RNAi plants (~0.8) (Burkart *et al.*, 2015). These data suggested that Spike is an important regulator of ROP function in polarized growth. However, the presence of protonemal extensions suggested that the *Spike*-RNAi phenotype was less penetrant than the *ROP*-RNAi phenotype, which could be due to the presence of ROPGEFs. Transformation with the *ROPGEF*-RNAi construct also resulted in small plants; however, solidity measurements demonstrated that these plants were significantly more polarized than those of *Spike*-RNAi (Fig. 2C). In fact, with the exception of two outliers, the majority of the plants had solidity values closer to the values of the controls . We also counted the number of polarized extensions for each plant and found that *ROPGEF*-RNAi had 4.81 ± 2.3 (*n*=27 plants), significantly more than we observed for *Spike*-RNAi plants. Together, these data suggest that in contrast to Spike, ROPGEFs do not significantly contribute to regulating cell polarity. In addition, *ROPGEF*-RNAi plants were more difficult to recover, and we were only able to image nine silenced plants per transformation, suggesting that ROPGEF may play a more critical role in protoplast regeneration or plant viability.

In contrast to GEFs, GAPs function to inactivate ROP by activating the intrinsic GTPase activity and generating a population of GDP-bound ROP. Therefore, loss of GAP function should lead to an accumulation of ROP-GTP, and thus a larger population of active ROP. Unfortunately, predicting the phenotype due to excess rather than reduced ROP function is more challenging. Using a heat-shock promoter, Ito *et al.* (2014) demonstrated that elevated levels of ROP or ROPGEF, which probably lead to higher ROP activity, results in apical swelling in protonemata, suggesting that polarized growth is impaired with too much ROP activity. However, Burkart *et al.* (2015) were not able to recover plants constitutively overexpressing ROP. Thus, it is possible that constitutive loss of GAP function, which would lead to excess ROP activity, may be lethal. We predicted that silencing the GAP proteins that mediate ROP function during protonemal growth would either lead to small plants with significant defects in polarity, or it would be difficult to recover actively silencing plants.

*Physcomitrella patens* has seven GAPs: six ROPGAPs and one REN. To determine which family of ROPGAPs regulates ROP activity during protonemal growth, we generated RNAi constructs to silence either the six ROPGAP genes or the single REN gene. The *ROPGAP*-RNAi construct contained ~200-bp regions of sequence from each of the six ROPGAP genes, and the *REN*-RNAi construct contained the first 423 bp of the REN coding sequence. We found that silencing ROPGAPs resulted in very small plants, only 18% the size of control RNAi plants (Fig. 2). The *ROPGAP*-RNAi plants had one or two protonemal extensions emanating from the initial protoplast, which was reflected in the high solidity values as compared to control RNAi plants. Additionally, we only recovered 14.7 *ROPGAP*-RNAi plants per transformation, suggesting that loss of ROPGAP activity is either incompatible with protoplast regeneration or is lethal, similar to what has previously been observed for overexpression of ROP (Burkart *et al.*, 2015). Interestingly, loss of REN function did not have a dramatic effect on protonemata (Fig. 2A). *REN*-RNAi plants were only 20% smaller than control plants (Fig. 2B) but they were still highly polarized, as indicated by the low solidity values (Fig. 2C). Taken together, these data suggest that ROPGAPs, rather than REN, play a predominate role in regulating ROP activity during protonemal growth.

To provide additional evidence that ROPGAPs regulate ROP activity, we used a directed yeast two-hybrid assay and found that five of the six ROPGAPs weakly interacted with ROP. As expected, ROPGAP did not interact with the dominant negative ROP but did interact strongly with the constitutively active version (Table 1). In contrast, REN exhibited no interactions with any form of ROP. These data suggest that ROPGAPs, and not REN, physically associate with the active form of ROP, providing corroborating data to support the hypothesis that ROPGAPs have a predominant role in regulating ROP activity during polarized growth.

**Table 1.** Summary of yeast two-hybrid results

	ROP3	ROP3 CA	ROP3 DN	ROP4	ROP4 CA	ROP4
ROPGAP1	–	+	–	–	+	–
ROPGAP2	+	+	–	+	+	–
ROPGAP3	+	+	–	+	+	–
ROPGAP5	–	–	–	–	+	–
ROPGAP6	–	+	–	+	+	–
REN	–	–	–	–	–	–

+ Indicates blue colonies grown on –Leu–Trp–His–Ade+AbA+X-α-gal.

– Indicates no growth on –Leu–Trp–His–Ade+AbA+X-α-gal.

Like the ROPGAPs, GDIs ultimately play an inhibitory role in ROP regulation. In contrast to ROPGAPS, GDIs inhibit ROP by removing GDP-bound ROP from the plasma membrane (Dovas and Couchman, 2005). Thus, loss of GDI activity should result in the accumulation of inactive ROP on the membrane, and most likely lead to a phenotype similar to loss of ROP function. Additionally, lack of GDI has been shown to reduce ROP protein levels (Boulter *et al.*, 2010; Feng *et al.*, 2016). In either scenario, silencing ROPGDI should reduce ROP signaling. The *P. patens* genome encodes four nearly identical ROPGDIs. The GDI1 (Pp3c3_32980) and GDI4 genes (Pp3c3_33010) differ by a single nucleotide (T510C). Similarly, the GDI2 (Pp3c10_19740) and GDI3 (Pp3c10_19650) genes also differ by only one nucleotide (G378A). Neither nucleotide change results in a change in the amino acid sequence, such that GDI1 and GDI4 encode the same protein, as do GDI2 and GDI3. Between the two protein sequences, GDI1/4 shares 80.95% sequence identity with GDI2/3, with the majority of divergence occurring within the first ~50 amino acids.

To silence the four ROPGDIs, we used a 408-bp fragment from the GDI2 cDNA to target all four genes. Transformation with the *GDI*-RNAi construct resulted in small plants, on average 48% the size of controls. Some plants phenocopied *ROP*-RNAi plants (Fig. 2A) (Burkart *et al.*, 2015). However, others had a number of unpolarized cells in the center of the regenerating plant with a few polarized extensions, while others were significantly more polarized. On average, the *ROPGDI*-RNAi plants were larger than the *Spike*-RNAi plants, but their solidity values were similar, reflecting a significant fraction of plants with a polarity phenotype. These data suggest that ROPGDIs play an important role regulating ROP function during polarized growth.

### A single GDI protein is sufficient for polarized growth

**Fig. 3.**
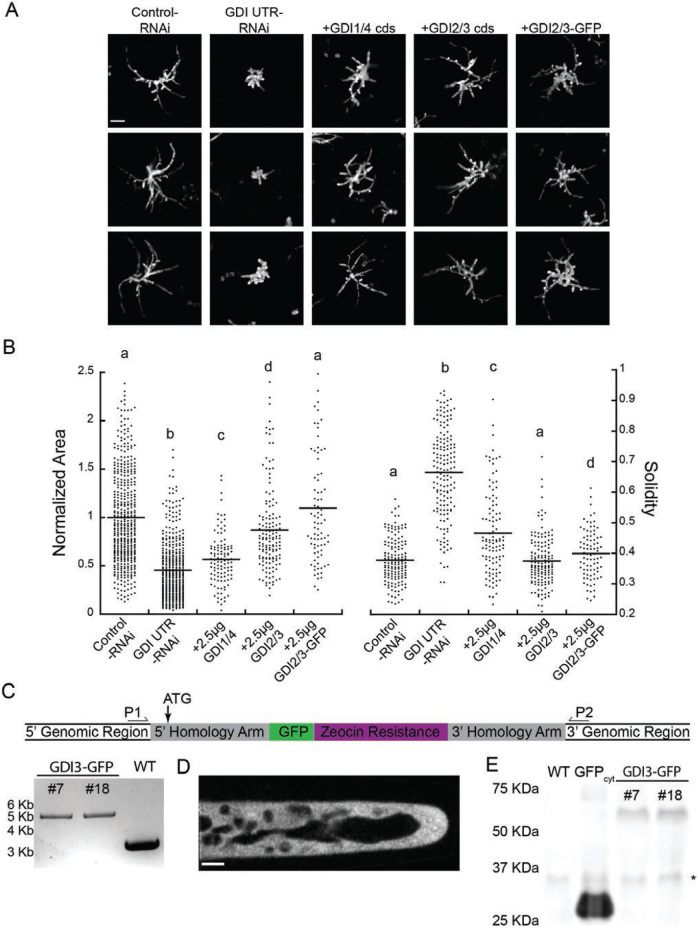
RopGDI2/3 rescues the *RopGDI*-RNAi phenotype. (A) Representative images of chlorophyll autofluorescence of 7-d-old plants expressing the indicated constructs. Complementation was performed by transforming 30 μg of the GDI UTR RNAi plasmid together with 2.5 μg of the indicated plasmid. The scale bar is 100 μm. (B) Quantification of plant area and solidity. Area is normalized to the average size of plants transformed with the control plasmid, and the means are indicated by horizontal lines. Different letters indicate groups with significantly different means as determined by ANOVA with a Tukey *post hoc* test (α=0.05). (C) Diagram of the modified GDI3-mEGFP locus (not to scale), with location of genotyping primers and start codon (ATG) indicated, And the results of genotyping the modified locus in two independent stable transgenic lines together with a wildtype (WT) control. An amplicon produced across the modified locus is 5620 bp, compared to 3339 bp in the WT. (D) Single-focal-plane confocal image of GDI3-mEGFP localization. The scale bar is 5 μm. (E) Western blot of GDI3-mEGFP lines, with WT and cytosolic GFP extracts as controls. The asterisk indicates a non-specific band detected with the GFP primary antibody.

Because the GDI genes share such high sequence similarity, there are essentially only two GDI proteins expressed in the cell. To determine if the two proteins are functionally redundant, we tested whether either one could rescue the RNAi phenotype. However, since the coding-sequence RNAi construct effectively silenced all four genes it would also silence a rescuing construct. Thus, we generated a second *GDI*-RNAi construct that targeted the 5′-UTRs of the *ROP*GDIs. The RNAi construct contained 300 bp of the 5′-UTRs immediately upstream of the start codon of GDI1, 2, and 3. The 5′-UTR of GDI4 had a 186-bp region that was 100% identical to GDI1, probably sufficient to silence GDI4. We found that 7-d-old plants expressing the UTR-based RNAi construct had a striking loss of polarity phenotype (Fig. 3A). On average, plants were 45% the size of control plants (Fig. 3B), similar to that observed with the coding sequence-based construct. However, the average solidity value was 0.67, which was a 1.3-fold increase from the coding-sequence construct (Fig. 2C) and a 1.76-fold increase from the control (Fig. 3B). These data suggest that the UTR-based construct more efficiently silenced the four GDI genes.

To test whether GDI1/4 or GDI2/3 could rescue the RNAi phenotype, we co-transformed the GDI UTR RNAi construct with a construct that constitutively expressed either the coding sequence of GDI1/4 or GDI2/3. Importantly, transcripts produced from these constructs did not contain the 5′-UTR, and thus could not be targeted by the UTR RNAi construct. Upon co-transformation of the UTR-based RNAi construct with 2.5 μg of either GDI1/4 or GDI2/3, we discovered that the two constructs exhibited different degrees of complementation (Fig. 3A, B). GDI2/3 largely restored normal growth; plants were on average 87% the size of control and the solidity of rescued plants was the same as that of the control (Fig. 3B). In contrast, GDI1/4 produced plants that were only 56% the size of control plants, with a solidity value closer to the control than to the UTR RNAi values (Fig. 3B), suggesting that polarity was rescued more than plant size. Together, these data indicate that GDI2/3 may play a more predominane role than GDI1/4 in polarized growth.

The transient complementation test also allowed us to examine the functionality of an in-frame GFP fusion of GDI2/3. When 2.5 μg of GDI2-mEGFP was co-transformed with the UTR-based RNAi construct, plants were 10% larger than controls, with a similar solidity value (Fig. 3A, B). Interestingly, the mEGFP fusion completely compensated for the silencing of all the endogenous GDIs, demonstrating that GDI2-mEGFP is sufficient to restore polarized growth and is a functional protein.

To investigate the localization of GDI2/3, we chose to tag GDI3 at the endogenous locus with an in-frame mEGFP via homologous recombination. We obtained two independent lines with a C-terminal mEGFP successfully integrated into the GDI3 locus (Fig. 3C). Using confocal microscopy, we observed that GDI3-mEGFP localized to the cytoplasm, similar to reports of GDI localization in other plants (Klahre *et al.*, 2006) and in yeast (Koch *et al.*, 1997) (Fig. 3D). To ensure that we observed GDI3-mEGFP, and not proteolytically cleaved mEGFP, we performed a western blot using plant extracts (Fig. 3E). A band corresponding to the molecular weight of GDI3-mEGFP (53kDa) was present in two GDI3-mEGFP tagged lines, but not in the wildtype or cytosolic mEGFP tissue (Fig. 3E). Therefore, we are confident that GDI3 was cytosolic under standard imaging conditions.

### Silencing ROPGDI stimulates actin dynamics

**Fig. 4.**
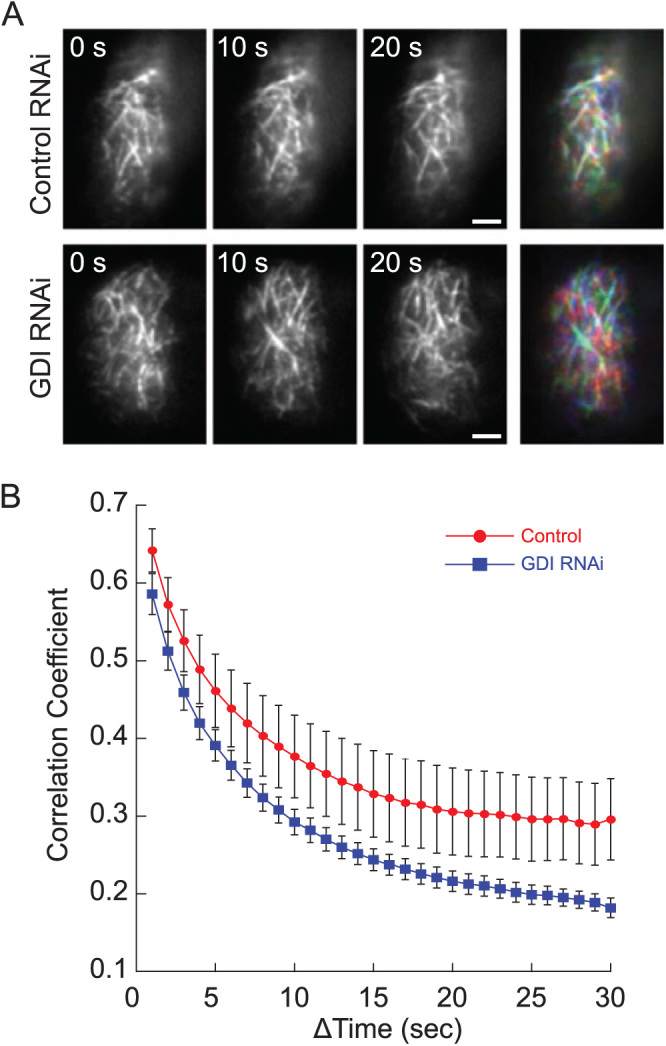
Silencing GDIs increases actin dynamics. (A) Representative images of cortical actin visualized by Lifeact-mEGFP. The scale bars are 2 μm. The merged images at the right show all three time points as separate color channels in a red, green, blue (RGB) image. More color indicates greater changes between frames, illustrating that the actin array changes more in *GDI*-RNAi than in control-RNAi cells. See Supplementary Movies S1 and S2. (B) The correlation coefficient of cortical actin decays faster in *GDI*-RNAi plants than in control-RNAi plants, indicating that the former have increased actin dynamics. Data are means (±s.e.m.): *GDI*-RNAi, *n*=14 cells; control-RNAi, *n*=11 cells.

If ROPGDI function is primarily mediated through ROP, then we would expect that silencing GDI would increase the pool of inactive ROP bound to the membrane. Since loss of ROP has been shown to stimulate actin dynamics at the cortex (Burkart *et al.*, 2015), we reasoned that silencing GDI should similarly result in an increase in cortical actin dynamics. To test this, we used the UTR-based RNAi construct to silence ROPGDIs in an NLS4 line that stably expresses the live-cell actin probe, Lifeact-mEGFP (Vidali *et al.*, 2009*a*). We identified actively silenced plants and used variable angle epifluorescence microscopy (VAEM) to image actin over time at the cell cortex (Fig. 4, Supplementary Movies 1, S2). To quantify the rate of changes in actin organization in the ROPGDI-silenced and control plants, we determined the correlation coefficient between images in the time-lapse acquisition and then plotted it for all temporal intervals. As the temporal interval increases, the correlation coefficient decays because more time has passed between images and the actin has reorganized. If the reorganization occurs more rapidly, then a steeper decay is observed. Using this analysis, we found that silencing ROPGDI, similar to silencing ROP (Burkart *et al.*, 2015), stimulated dynamic cortical actin rearrangements (Fig. 4). These data suggest that GDI functions through ROP to regulate polarized cell expansion.

## Discussion

ROPs are described as the master regulators of tip growth in plants (Feiguelman *et al.*, 2018), yet much remains to be elucidated about their regulation. ROP regulators are subject to three main types of regulation: activation by ROPGEFs, inactivation by ROPGAPs, and sequestration by ROPGDIs. In addition, in plants there are proteins with GEF domains, the Spikes, and proteins with GAP domains, the RENs. Here, we systematically studied the contribution of each family to tip growth in the model moss, *P. patens*. In addition to regulators, ROPs have known downstream effectors, the RIC proteins, only one of which is encoded within the *P. patens* genome. We demonstrated that the loss of *PpRIC1* was not detrimental to protonemal growth or regeneration from protoplasts (Fig. 1). In fact, *RIC*-RNAi plants were larger on average. Interestingly, this mirrors Arabidopsis *ric1* pollen tubes, which grow faster than controls. Conversely, *AtRIC1* over-expression inhibits pollen tube germination and growth (Zhou *et al.*, 2015).

In contrast to the RIC-silencing phenotype, we demonstrated that there was a differential inhibitory effect on plant morphology in the absence of each family of ROP regulators. For instance, protonemal growth was severely inhibited with reduced ROPGAP function. However, the loss of the other GAP protein, REN, was far less deleterious (Fig. 2). In addition to plant size, silencing ROPGAP produced plants with higher solidity than the controls, which we interpret as a reduction in plant polarity. Silencing REN had fewer adverse effects on plant size and polarity than the loss of ROPGAPs, suggesting that either the ROPGAPs have a more significant role in the regulation of ROP in *P. patens* or that REN was not sufficiently silenced in plants expressing the *REN*-RNAi plasmids. In support of the former possibility, REN did not interact with any form of ROP in our yeast two-hybrid assays (Table 1). This is in stark contrast to Arabidopsis *ren1* pollen tubes, which exhibit dramatic tip ballooning (Hwang *et al.*, 2008). Interestingly, Arabidopsis *gap1,3* pollen tubes grow at rates similar to the wildtype (Hwang *et al.*, 2010). Thus, in the 450 million years since their divergence (Rensing *et al.*, 2008), Arabidopsis may have evolved to utilize RENs as GTPase-activating proteins during tip growth, while *Physcomitrella* may have co-opted the ROPGAPs.

Interestingly, both families of GEFs had striking RNAi phenotypes (Fig. 2). Silencing each family of ROPGEFs or Spikes resulted in plants that were ~25% the size of controls. This is in contrast to previous observations in Arabidopsis. For example, *Δropgef1,9,12,14* quadruple-mutants have pollen tubes that are ~80% the length of controls (Chang *et al.*, 2013), a comparatively mild phenotype. The Arabidopsis genome only contains a single Spike, and *spk1* plants have irregularly shaped and under-branched trichomes (Basu *et al.*, 2008). Whether *AtSPK1* regulates ROP during pollen tube growth, thus compensating for *Δropgef1,9,12,14*, remains to be elucidated. However, given that both *ROPGEF*- and *Spike*-RNAi in *P. patens* led to a reduction in protometal growth, it suggests that both families of GEFs contribute to tip growth regulation in moss. Although the reduction in overall plant size was similar in *ROPGEF*- and *Spike*-RNAi, there were differences in morphology. *Spike*-RNAi plants often contained a cluster of spherical cells at their core, from which a few polarized extensions emanated (Fig. 2A). This phenomenon was not observed in *ROPGEF*-RNAi plants. This suggests that Spikes play a role in establishing polarity during protoplast regeneration, as the young plants are less able to develop polarized features early in the regeneration process.

Unlike the GAPs and GEFs with two gene families each in *P. patens*, ROPGDI is a single four-member gene family that encodes for two proteins. Silencing all four ROPGDIs resulted in plants that were less than half the size of control plants and had few polarized extensions (high solidity) (Fig. 2A, Fig. 3). Because ROPGDIs function to sequester the hydrophobic prenyl group of inactive ROP, thus making a cytosolic inactive heterodimer, cells lacking ROPGDIs probably have an accumulation of inactive ROP at the plasma membrane. As predicted, transformation of the *GDI*-RNAi construct resulted in plants that exhibited a similar phenotype to that observed when ROP levels were reduced (Burkart *et al.*, 2015).

Consistent with previous analyses of the actin cytoskeleton in ROP-silenced plants (Burkart *et al.*, 2015), we found that GDI-silencing, which inhibited polarized growth, resulted in an increase in actin dynamics (Fig. 4). These data corroborate the model that proper actin dynamics are a prerequisite for polarized growth (Gibbon *et al.*, 1999; Vidali *et al.*, 2001). In Arabidopsis, plants lacking *AtGDI1* have short, isotropic root hairs (Carol *et al.*, 2005). Similarly, pollen tubes with decreased *AtGDI2b* levels are thicker (less polar) than controls (Hwang *et al.*, 2010). Yet on the whole, Arabidopsis plants lacking all three ROPGDIs grow fairly well (Feng *et al.*, 2016), which is in contrast to the dramatic loss of polarized extensions in *P. patens* protonemata when the ROPGDIs were silenced. These observations suggest that ROPGDIs may not be explicitly required for diffuse cell growth and instead are more important in tip-growing cells.

In this study, we have established a systematic understanding of the relative contribution of each ROP regulator in controlling tip growth in *P. patens*. In contrast to seed plants, the *P. patens* genome contains only four, nearly identical, ROP genes. Thus, it is intriguing to speculate that mosses expanded the set of ROP regulators, as opposed to the number of ROP genes. The largely expanded families of ROP regulators might then be instrumental in specifying cell shape throughout development in mosses. Interestingly, a number of ROP regulators, such as RIC and REN, did not dramatically affect tip growth when transiently silenced. While incomplete silencing of these genes could account for the absence of a phenotype, it is also possible that these ROP regulators may play a more central role in other developmental stages. To address this, future studies investigating expression patterns of each gene family member together with null mutants of genes expressed in specific tissues are required to elucidate the roles ROP regulators may have beyond tip growth.

## Supplementary data

Supplementary data are available at *JXB* online.

Table S1. List of primers used in this study.

Movie S1. VAEM time lapse of Lifeact-mEGFP in control and *GDI*-RNAi cells (full details are given in the file containing Table S1).

Movie S2. Walking average of Movie S1 (full details are given in the file containing Table S1).

## Supplementary Material

Supplementary Table S1

Supplementary Movies S1

Supplementary Movies S2
