# Supplementary material for: Systematic survey of the function of ROP regulators and effectors during tip growth in the moss Physcomitrella patens
Source: J Exp Bot. 2018 Oct 31;70(2):447–57. doi: 10.1093/jxb/ery376 (PMC6322563; doi:10.1093/jxb/ery376)
Supplement: Supplementary Table S1 [file ery376_suppl_supplemenentary_table_s1.pdf]

SUPPLEMENTARY DATA

Movie S1. GDI knock down increases actin dynamics. A representative VAEM time lapse of Lifeact-mEGFP near the cortex in control (left) and GDI RNAi (right) cells.

Movie S2. Walking average of Movie 1. In the walking average, frames 1-4 (4 sec of real time) are averaged, then 2-5, then 3-6, etc. The averaged frames are then played back with 1 sec in between each averaged frame. The averaging helps to enhance the differences in dynamics between the control (left) and GDI RNAi plants (right).

Table S1. Primers used in this study.

ID	Name	Sequence	Purpose
2343	RopGAP2cdsiF_Bsu36 1	CACCCCTAAGGAACAAGGGCTCATGGCTG	GAP2 amplicon
23244	RopGAP2cdsiR_EcoRI	GAATTCGGCCCAGTTCAATAGAGCAGAC	GAP2 amplicon
2345	RopGAP6cdsiF_EcoRI	CACCGAATTCAGTGTTCCGACTATCCTAC	GAP6 amplicon
2346	RopGAP6cdsiR_Bsu36 1	CCTAAGGGTCGGCCATCTGGGTCAT	GAP6 amplicon
913	Gap60RNAiBamHI-F	CACCGGATCCGCGTGGTTTAGAGAACTTCCAAGAGG	GAP4 amplicon
914	Gap60RNAiBsu36I-R	CCTTAGGGTTTGGGGCAAACACCATAGCG	GAP4 amplicon
915	Gap159RNAiBsu36I-F	CACCCCTTAGGGCGTGGCTCGAGAACTTCCAACAGG	GAP5 amplicon
916	Gap159RNAiBamHI-R	GGATCCGCGTCATGTTAGGTGCAAACACCATTGC	GAP5 amplicon
828	GAP107-133941RNAi-F	CACCCTTCCAAGAGGCGTTCTAGATTCG	GAP3 amplicon
834	GAP107-133941BamRNAi-R	GGATCCCTGTGTCATGTTCGGAGCAAAAACC	GAP3 amplicon
829	GAP13-44811BamRNAi-F	CACCGGATCCTGGTTTCGGGAGTTGCCGC	GAP1 amplicon
830	GAP13-44811RNAi-R	TTAGGAGCAAAGACCATTGCAATATTGC	GAP1 amplicon
901	GEF28cdsRNAi BamHI-F	CACCGGATCCGCTTGAAAGCTACCATGAGACGG	GEF6 amplicon
902	GEF28cdsRNAi Bsu36I-R	CCTTAGGCTTGTGCATTGATAGCCATAGCAGC	GEF6 amplicon
903	GEF34cdsRNAi Bsu36I-F	CACCCCTTAGGCGGAATTCTGGTACGTGGACCACG	GEF5 amplicon
904	GEF34cdsRNAi BamHI-R	GGATCCCCATCTCCGACAAAACTTGCGCG	GEF5 amplicon
825	GEF58-42414RNAi-F	CACCACGGAATTCTGGTATGTGGACC	GEF2 amplicon
826	GEF58-42414BamRNAi-R	GGATCCTCCATCTCAACCAAAACTTGCGC	GEF2 amplicon
827	GEF22-22294RNAi-R	CTTCGGAAGCGAATCCCAATACAC	GEF4 amplicon
833	GEF22-22294BamRNAi-F	CACCGGATCCTCAAGGTTGTCTATGCAGAGACAAGAAG	GEF4 amplicon
1679	GEF7cdsiNotI-F	CACCGCGGCCGCCGTCAGTGTTTGGAGAGTTGTGG	GEF1 amplicon
1680	GEF7cdsiBamHI-R	GGATCCCCGTGCTCAACATACCAGAATTCC	GEF1 amplicon
1681	GEF283cdsiBamHI-F	CACCCGATCCCCTGAGGAACGACGAATGAGG	GEF3 amplicon
1682	GEF283cdsiNotI-R	GCGGCCGCCGACATGACAACGCTACGATCC	GEF3 amplicon
1817	Ren2-F	CACCATGTCAGCTGCAGTGAGCCCTG	REN amplicon

1820	Ren1 RNAi R	CCTTCGACTGGTCGCCTGAAACC	REN amplicon
1621	Spike145cdsi-F	CACCATGGGTGAGATTGCCTTAAGGTGG	SPK6 amplicon
1622	Spike145cdsiBamHI-R	GGATCCGCATCACTTCCTGTTGACCTCC	SPK6 amplicon
1623	Spike302cdsiBamHI-F	CACCGGATCCATGGCTTTAACCAGCATGGATTC	SPK2 amplicon
1624	Spike302cdsinNotI-R	GCGGCCGCCTTGCTGCAAGACGCAACT	SPK2 amplicon
1625	Spike60cdsiBamHI-F	CACCGGATCCCCAGTGTGCAGTTGCGTTGC	SPK3 amplicon
1626	Spike60cdsiBamHI-R	GGATCCCCTTGCAGTTCAGGCTGCC	SPK3 amplicon
1223	Spike1-318 RNAiBamHI-F	CACCGGATCCGAATGGATGGCGACCAGGCC	SPK1 amplicon
1224	Spike1-318 RNAi-R	CCTCTTCTCCAATTAACCTAGAATGAACGCG	SPK1 amplicon
1225	Spike1-25 RNAi-F	CACCCATGTGGGGCAAGGAACATGTGG	SPK5 amplicon
1226	Spike1-25 RNAiBamHI-R	GGATCCAGTTGTCCGTAGGCACGCCTTGC	SPK5 amplicon
1227	Spike1-59 RNAiBamHI-F	CACCGGATCCGAGGTCCTCGTCAACATTCAGTGC	SPK4 amplicon
1228	Spike1-59 RNAiBamHI-R	GGATCCGCTCTCCTGCTAAAGCTGCTGTACG	SPK4 amplicon
959	Ric5'utrcdsRNAiF	CTGAACAGATGTTAGACGCTCTG	RIC amplicon
212	RIC-CDS-RNAiR	CTGCATTGTTGAACGGCCTACTTG	RIC amplicon
899	ROPB CDS RNAi-F	CACCATGAGCACTTCACGGTTTATCAAGTGC	ROP4 Full-length coding sequence
533	Rop D FL F	CACCATGAGCACTTCTCGCTTTATCAAGTGC	ROP3 Full-length coding sequence
537	Rop D FL R	TTAGAGAATGACACAGTTCTTTTGCTTCTTTTTCTTC	ROP3/4 Full-length coding sequence
1380	RopB-G15V-F	GACTGTTGGAGATGTAGCTGTAGGGAAGACGTGC	ROP4 mutagenesis
1381	RopB-G15V-R	GCACGTCTTCCCTACAGCTACATCTCCAACAGTC	ROP4 mutagenesis
1382	RopB-T20N-F	GCTGTCGGGAAGAATTGTATGCTTATTTCATACACC	ROP4/3 mutagenesis
1382	RopB-T20N-R	GGTGTATGAAATAAGCATACAATTCTTCCCGACAGC	ROP4/3 mutagenesis
1814	ROPD-G15V-F	CAAGTGCGTTACTGTTGGAGATGTAGCTGTGGGGAAG AC	ROP3 mutagenesis
1815	ROPD-G15V-R	GTCTTCCCCACAGCTACATCTCCAACAGTAACGCACTTG	ROP3 mutagenesis
1817	Ren2-F	CACCATGTCAGCTGCAGTGAGCCCTG	REN FL cDNA
1819	Ren1-R	TCAAGGGCTTCTCTTCGTTGGGG	REN FL cDNA
1165	CACC-GAP256F	CACCATGACAGAGGTTCTCCCAACCC	GAP6 FL cDNA
1166	GAP256R	CTATCCTCCACACGTACCAGACAC	GAP6 FL cDNA
1737	PpGAP13-44811-F	CACCATGACACAGGTTCTCCCGACC	GAP1 FL cDNA
1738	PpGAP13-44811-R	CTACCAAGCTTCTACCCTTTGATTGTTG	GAP1 FL cDNA
1739	PpGAP107-133941-F	CACCATGAGCTTGGATATTGGATGGCC	GAP3 partial cDNA
1754	PpGAP107-133941-R	TCACCAGGCTTCCACACGTTC	GAP3 partial cDNA
1740	PpGAP159-14749-F	CACCATGGATATTGGGTGGCCAACAG	GAP5 partial cDNA
812	PpGAP60-45234-R	TCACCATGCTTCCACCCGTTC	GAP2, and GAP5 partial cDNA
1736	PpGAP1-23485-F	CACCATGGAAATTGGATGGCCGACG	GAP2 partial cDNA

1764	PpRopGDI-cdsi-F	CACCGTGGAGCCGGAGGTGAACG	GDI cds RNAi construct
1765	PpRopGDI-FL-cdsi-R	CTACCACTCCTTCCGGATCTCGAACG	GDI cds RNAi construct & rev primer for all GDI full length cDNAs
1766	PpRopGDI-1-flcds-F	CACCATGTCGAAGGCGATGGCCG	GDI1 full-length cDNA
1767	PpRopGDI-2-3-flcds-F	CACCATGTCGAAGACTGTAGCTGGGTGG	GDI2/3 full-length cDNA
1915	GDI1_5.UTR_F	CCACCAGCCACCCCGTCGCC	GDI 5'UTR RNAi construct
1926	GDI1_5'UTR_R 3.0	TCTTGGATCCCTTTCGACCTCCCCAC	GDI 5'UTR RNAi construct
1917	GDI2_5.UTR_F	CACCGGATCCACCCTTGCATCGCCAT	GDI 5'UTR RNAi construct
1927	GDI2_5'URT_R 3.0	TCTTGAATTCCCCTTCCACCCCGAGAT	GDI 5'UTR RNAi construct
1913	GDI3_5.UTR_F	CACCGAATTCACCCGAAAACATTTCG	GDI 5'UTR RNAi construct
1923	GDI3_5.UTR_R 2.0	CCTTCCACCCCGAGATTCT	GDI 5'UTR RNAi construct
1972	attB1RopGDI2F	GGGGACAAGTTTGTACAAAAAAGCAGGCTTT ATGTCGAAGACTGTAGCTGG	GDI2-GFP fusion
1973	attB5rRopGDIR	GGGGACAACTTTTGTATACAAAGTTGTCCACTCCTTCCG GATCTCGA	GDI1/2-GFP fusion
1974	attB1RopGDI1F	GGGGACAAGTTTGTACAAAAAAGCAGGCTTTATGTCGA AGGCGATGGCCGG	GD1-GFP fusion
1991	5'TargetArmGDI3attB 1	GGGGACAAGTTTGTACAAAAAAGCAGGCTATTTAAATA AACGAATGTGCTGTCAGAT	GDI3-GFP knock-in construct
1992	5'TargetArmGDI3attB 5r	GGGGACAACTTTTGTATACAAAGTTGTCCACTCCTTCCG GATCTCGAA	GDI3-GFP knock-in construct
1993	3'TargetArmGDI3attB 3	GGGGACAACTTTGTATAATAAAGTTGATATAGATCCCG AGTCGGCA	GDI3-GFP knock-in construct
1994	3'TargetArmGDI3attB 2	GGGGACCACTTTGTACAAGAAAGCTGGGTATTTAAATT TACTCCTCTTTTAGGCAAC	GDI3-GFP knock-in construct
